# The Source, Spatial Distribution and Risk Assessment of Heavy Metals in Soil from the Pearl River Delta Based on the National Multi-Purpose Regional Geochemical Survey

**DOI:** 10.1371/journal.pone.0132040

**Published:** 2015-07-31

**Authors:** Lingyan Zhang, Shuhai Guo, Bo Wu

**Affiliations:** 1 Institute of Applied Ecology, Chinese Academy of Sciences, Shenyang, 110016, China; 2 University of Chinese Academy of Sciences, Beijing, 100049, China; Sun Yat-Sen University, CHINA

## Abstract

The data on the heavy metal content at different soil depths derived from a multi-purpose regional geochemical survey in the Pearl River Delta (PRD) were analyzed using ArcGIS 10.0. By comparing their spatial distributions and areas, the sources of heavy metals (Cd, Hg, As and Pb) were quantitatively identified and explored. Netted measuring points at 25 ×25 km were set over the entire PRD according to the geochemical maps. Based on the calculation data obtained from different soil depths, the concentrations of As and Cd in a large area of the PRD exceeded the National Second-class Standard. The spatial disparity of the geometric centers in the surface soil and deep soil showed that As in the surface soil mainly came from parent materials, while Cd had high consistency in different soil profiles because of deposition in the soil forming process. The migration of Cd also resulted in a considerable ecological risk to the Beijiang and Xijiang River watershed. The potential ecological risk index followed the order Cd ≥ Hg > Pb > As. According to the sources, the distribution trends and the characteristics of heavy metals in the soil from the perspective of the whole area, the Cd pollution should be repaired, especially in the upper reaches of the Xijiang and Beijiang watershed to prevent risk explosion while the pollution of Hg and Pb should be controlled in areas with intense human activity, and supervision during production should be strengthened to maintain the ecological balance of As.

## Introduction

The Pearl River Delta (PRD), located at the southern end of the Nanling metallogenic belt, is a very complicated large-scale estuarine system in China. The delta was formed and maintained by the deposition of sediments where the Pearl River flows into the sea. As a result of the substantial supply of heavy metals from the industrial, commercial and domestic activity of the region [1,2], the soil in the PRD differed greatly from what would otherwise be its natural soil type. The Pearl River consists of two main branches, the Xijiang and the Beijiang Rivers, both of which receive a high load of heavy metals annually. The well-developed river network and tidal action indicate that the characteristics of the heavy metals presented in the soil, as well as their associated chemical processes, are complex [3]. The PRD has experienced rapid economic growth in the past three decades. The main industries in the PRD region include electronics, textiles, paper making and cement production, which could be sources of heavy metals in soil. The complicate natural condition combined with strong anthropogenic activities made the PRD region a typical unit for the source research of heavy metal.

Previous studies have concluded that in general, the parent material is a critical factor in determining the type and concentration of magnetic minerals [4,5]. Other natural factors, such as the water regime and atmospheric transmission, could contribute to various heavy metal characteristics in soils [6,7]. In addition, the range of anthropogenic heavy metal sources has become more complex in recent decades, including the growth of metalliferous mining and smelting, the chemical industry, fossil fuel combustion, waste incineration, and agricultural activities [8–10]. Heavy metals also have distinct vertical distributions in the soil, especially around mining or metallurgical sites. Godin [11] attempted to evaluate the downward migration of metals and noted that the Cd, Cu, Hg, Pb and Zn content decreased logarithmically with depth. Sterckeman *et al*. [12] further quantified the vertical distribution of Cd, Pb and Zn at depths of 1.5–2.0 m according to the characteristics of heavy metals and soils.

A number of studies on the heavy metal contamination of surface soil in the PRD have been conducted. These studies reported that the rapid industrial development and urbanization over the last few decades has considerably increased the enrichment of heavy metals in the soil of the PRD. Bai and Liu [13] confirmed that the rapid development of electronics and electroplating industries is highlighted as the main cause of the increase in the concentration of heavy metals in the soil of the PRD in recent decades, and the degree of heavy metal pollution in soils had decreased in the following order: Cd > Cu > Ni > Zn > As > Cr > Hg > Pb. Bian *et al*. [14] found that the agricultural soil of the PRD was strongly influenced by cultivation methods, as well as human activities such as industrial development and the associated emission of pollutants.

To date, there have been very few studies on different soil profiles of the PRD region. Research that compares the level of contamination in the upper and lower layers of PRD soil, which might be an effective method for diagnosing the sources of heavy metals [12], is scarce. The accumulation and deposition of heavy metals in the PRD region might be affected by different mechanical and chemical processes. While challenging, it is essential to determine the sources of heavy metals in the PRD, where totally unpolluted soils are almost impossible to find. Since 1999, the National Multi-Purpose Regional Geochemical Survey (NMPRGS) has been performed in the agriculturally and industrially developed regions of China by the China Geological Survey [15], involving detailed investigation of heavy metals in the top and deep layers of soil. The PRD has been a major area of focus for the NMPRGS. Accordingly, based on the results of the NMPRGS, the aims of the present study were to:

- Analyze the causes of heavy metals in the PRD region and judge the level of contamination.
- Develop a method for diagnosing the severity of heavy metal pollution via comparison of top/deep layer soil with some crucial parameters.

## Materials and Methods

### Study area and data sources

**Fig 1:**
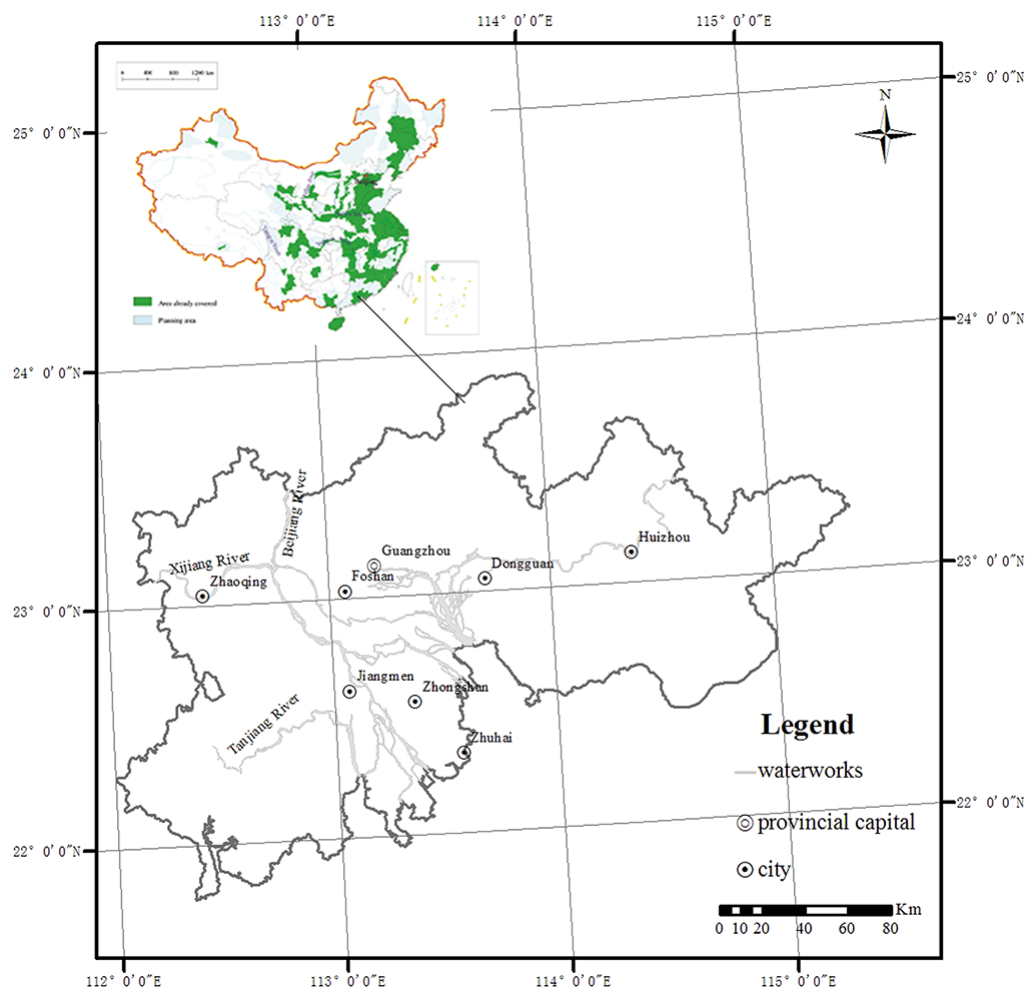
A major area of focus for the NMPRGS [16], the PRD region.

This study was performed in the PRD of Guangdong Province, China (Fig 1) from 21°43′N to 23°56′N, 112°00′E to 115°24′E. Published data were quoted from the National Multi-Purpose Regional Geochemical Survey (the part of the Pearl River Delta of Guangdong Province, China), which was performed by the Ministry of Land and Resources of China. Additionally, the field studies the Pearl River Delta of Guangdong Province, China did not involve endangered or protected species. The PRD was characterized as a quintessential subtropical monsoon climate. It is an extremely complex and large-scale estuarine system that consists of a tidal river network. The Xijiang and Beijiang Rivers are the two major river systems of the Pearl River Basin, which converge in Foshan City. The land is heavily industrialized and has been extensively cultivated in the flat parts of the region.

**Fig 2:**
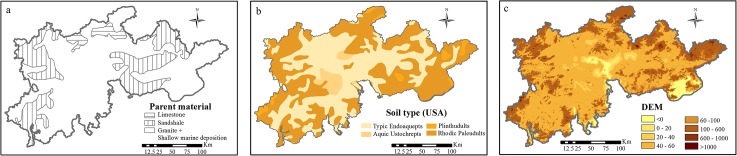
Basic message of the PRD region (a), geological sketch indicating the distribution of parent rocks (b), soil type and (c) DEM.

The PRD soil profile is the typical Al-enriched weathering profile and the product of the latest stage of weathering. It mainly developed from granite and shallow maine deposition, with sandshale and limestone (Fig 2A). Because of the intensive eluviation and illuviation with the hydrothermal conditions of the study area, the soil profiles were deficient in soluble salt, alkali metal, and alkali-earth metal, but they were rich in Fe and Al oxides and H^+^ [17]. As the downthrow of the region, low- elevation hills developed.

All sampling data were obtained from the Multi-Purpose Regional Geochemical Atlas, the Pearl River Delta economic zone in Guangdong Province, sampled from 2005 to 2009 [16]. To produce these data, a geochemical measurement with two-layer grid sampling was employed. Topsoil samples at depths of 0–20 cm were taken with a density of 1 sample per 4 km^2^. These samples were collected with 3 to 5 points around the corresponding sampling spot to represent the main soil type of each grid. The deeper soil samples (150–200 cm) were generally collected at the center of each 16 km^2^ grid. Hill landforms distributed around the PRD mainly had a lower elevation, as observed from the DEM. When the soil depth did not reach 150–200 cm, a proper site was set near to the original sampling spot. A total of 10667 surface soil samples and 2751 deep soil samples were collected with the method of two-layer gridding sampling in the study area by National Multi-Purpose Regional Geochemical Survey [16,17].

The external and internal quality controls were combined to control the analyzed quality of soil and coastal sediment samples. External quality control was performed by analyzing 4 pieces of blind control samples for each group of 50 samples, and there were a total of 1327 pieces. Internal quality control was performed by analyzing 4 pieces of certified reference materials for each group of 50 samples along with the collected samples. The average qualification rate of internal inspection was above 99.8% [16].

The reused figures were initially published under an open-access license.

### Statistical analysis

For statistical analysis, the data from the superficial and deep layers were treated separately. The top layer contained most of the anthropogenic input, while the deep layer represented the lithogenic input, with only minor anthropogenic contamination.

#### (i) Graphic processing

Geochemical maps of the PRD were vectored in ArcGIS 10.0 and re-sampled using a trial-and-error method to form the new raster with kriging interpolation. The distributions of high-concentration metals in the surface and deep soil were compared. Each original map was segmented into 4 to 6 pieces according to the density of coloring grades and it was then re-valued with a different color. The grid spacing was the same as the original spacing of data and the search radius was 2.5 times of the grid spacing. The index factor was 5 and the interpolation scheme was the inverse distance weighing. If the areas of the original and the new spots were not significantly different (p < 0.05), the new spots were accepted.

#### (ⅱ) Spatial disparity

If heavy metals in the surface and deep soil were from the same source, it was considered that the geometric center of high concentration areas should be coincident or adjacent to each other. Adjacent geometric center points in the surface soil and deep soil were paired and their spatial disparity was calculated by using the equation based on Euclidean Distance, which was the basic geometric distance formula for point to point as follows:

$$d=\frac{1}{n}•\sum_{i=1,j=1}^{n}\sqrt{{\left(x_{i}-x_{j}\right)}^{2}+{\left(y_{i}-y_{j}\right)}^{2}}\;,$$ (1)

where *n* was the paired number of geometric center points in the surface soil and deep soil (i.e., three); (*x*_*i*_, *y*_*i*_) and (*x*_*j*_, *y*_*j*_) represented the spatial coordinates of paired geometric center points in surface and deep soil; *d* was the average distance of the geometric center between the surface and deep soil. The larger the value of *d*, the smaller the correlation between the surface and deep soil. Points without matching were ignored.

#### (ⅲ) Potential ecological risk assessment

Netted measuring points at 25 × 25 km were set over the entire PRD and assigned values according to the geochemical maps. The potential ecological risk index (PER) was applied to assess the degree of heavy metal contamination as follows:

$$\text{Cd}:\;{\text{C}}_{\text{f}}^{\text{i}}={\text{C}}^{\text{i}}\;/\;{\text{C}}_{\text{n}}^{\text{i}}={\text{C}}^{\text{i}}\;/\;0.056,{\text{E}}_{\text{r}}^{\text{i}}={\text{T}}_{\text{r}}^{\text{i}}\times {\text{C}}_{\text{f}}^{\text{i}}=20\times {\text{C}}^{\text{i}}\;/\;0.056$$ (2)

$$Hg\;:\;C_{f}^{i}=C^{i}\;/\;C_{n}^{i}=C^{i}\;/\;0.078,E_{r}^{i}=T_{r}^{i}\times C_{f}^{i}=28\times C^{i}\;/\;0.078$$ (3)

$$As\;:\;C_{f}^{i}=C^{i}\;/\;C_{n}^{i}=C^{i}\;/\;8.9,E_{r}^{i}=T_{r}^{i}\times C_{f}^{i}=10\times C^{i}\;/\;8.9$$ (4)

$$Pb\;:\;C_{f}^{i}=C^{i}\;/\;C_{n}^{i}=C^{i}\;/\;36,E_{r}^{i}=T_{r}^{i}\times C_{f}^{i}=5\times C^{i}\;/\;36$$ (5)

$$RI=\sum E_{r}^{i}$$ (6)

where $C_{f}^{i}$ was the single element pollution factor, *C* ^*i*^ was the concentration of the element in samples, and $C_{n}^{i}$ was the reference value of the element. In this study, the $C_{n}^{i}$ values for Cd, Hg, As and Pb were 0.056, 0.078, 8.9 and 36 mg kg^-1^ according to the background value of the Guangdong Province [18]. The sum of $C_{f}^{i}$ for all metals examined represented the integrated degree of environmental pollution. $E_{r}^{i}$was the potential ecological risk index of an individual element. $T_{r}^{i}$was the biological toxic factor of an individual element, defined as 20 (for Cd), 28 (for Hg), 10 (for As), and 5 (for Pb) [19]. RI was the comprehensive potential ecological index, which was the sum of *E* ^*i*^_*r*_, where RI < 150 indicated low ecological risk, 150 < RI < 300 was moderate ecological risk, 300 < RI < 600 was considerable ecological risk, and RI > 600 was high ecological risk.

## Results and Discussion

### Spatial distribution of heavy metals

**Fig 3:**
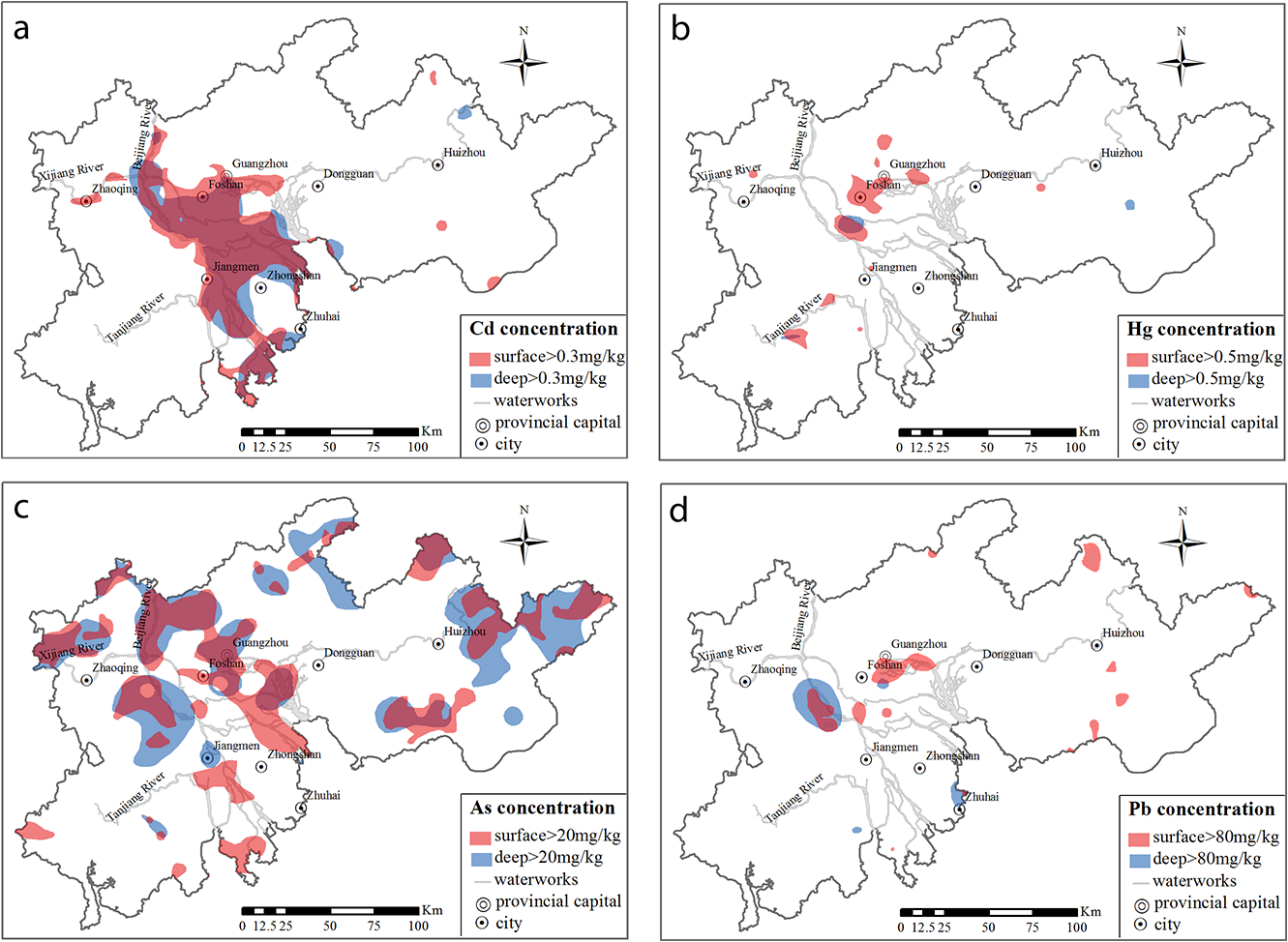
Distribution of high-concentration heavy metals in the soil of the PRD. (a) Cd; (b) Hg; (c) As; and (d) Pb.

The spatial distributions of heavy metals with high concentrations were compared between the surface soil and deep soil (Fig 3). Concentrations of heavy metals above the national second-class standard for soil of the People's Republic of China (GB15618-1995) were considered high in this study (the threshold concentration of Pb was set as 80 mg kg^-1^ according to soil pollution assessment technical regulations, which were revised on the basis of GB15618-1995 by the Ministry of Environmental Protection of the People's Republic of China).

#### Cd

**Fig 4:**
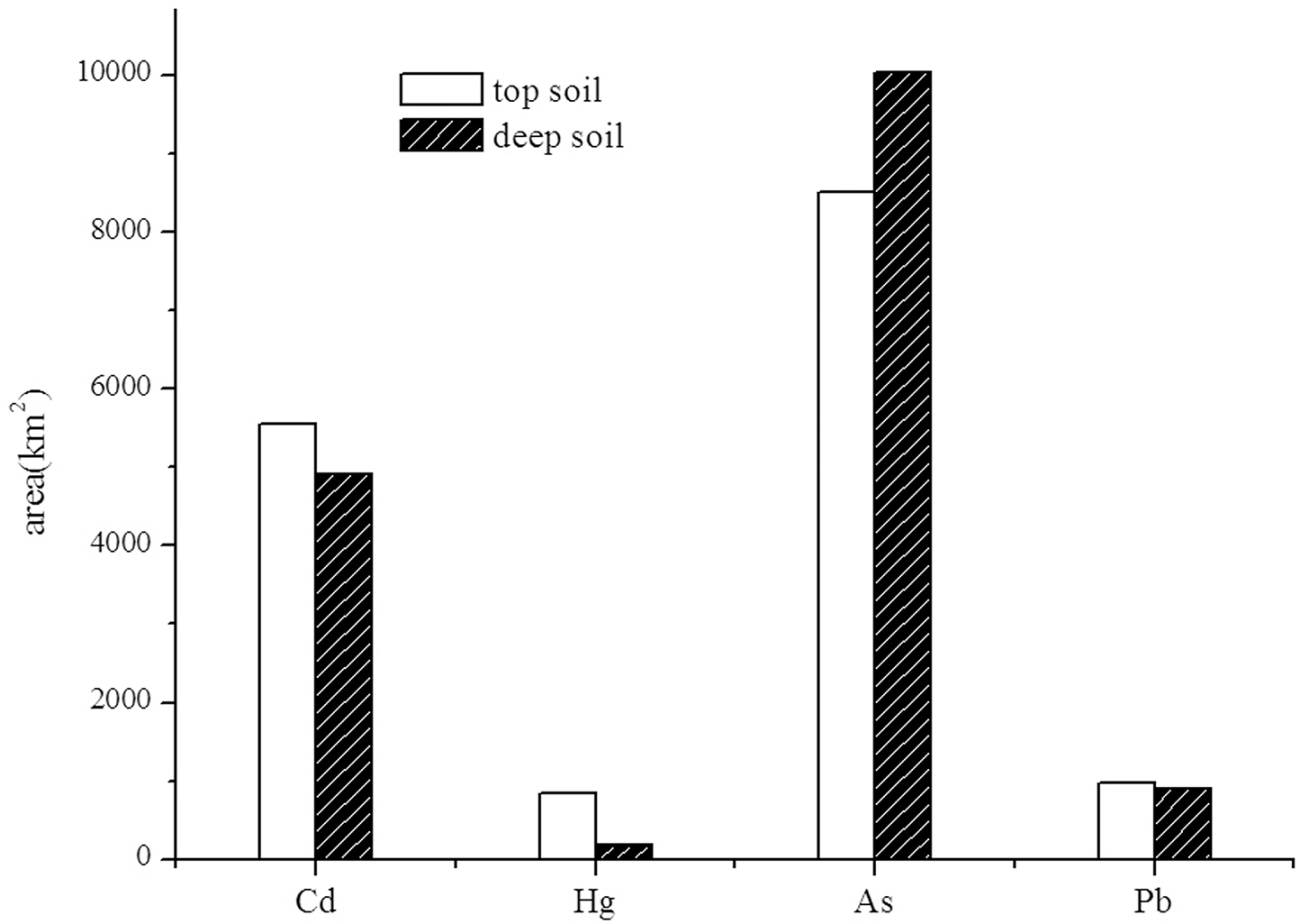
Areas of high-concentration metals above GB15618-1995 in soil.

A large area was contaminated by a high Cd concentration (> 0.3 mg kg^−1^) in both the surface and deep soils (Fig 3A), accounting for 13.3% and 11.8% of the PRD, respectively (Fig 4). These areas were almost coincident with the watersheds of the Beijiang and Xijiang Rivers. Cd had the largest coefficient of variation among the four heavy metals, both in the surface soil (410%) and in the deep soil (247%). This indicates that the concentration of Cd in the PRD differed to a greater extent than the other three metals. The area of Cd with a concentration of > 0.3 mg kg^−1^ was larger in the surface soil than that in deep soil, and it was spatially consistent. As an typical alluvial plain, soil in the PRD region formed under the effect of river sedimentation. The oscillation of flow caused by the regular tidal action in the Xijiang and Beijiang Rivers was advantageous to the deposition of Cd in the PRD area [3], and it aggravated the accumulation in the sediment. According to the large scale investigation of a series of alluvial plains by Cheng et al. [20], suspended substance carrying high levels of Cd was transported along with water and gradually deposited in the soil. Therefore, Cd in the soil vertical profile was mostly stable. Only in recent decades has the Cd content increased from anthropogenic input. The PRD region was a typical alluvial plain. Because of the intensive eluviation and illuviation in the hydrothermal conditions of the study area, the soil profiles were rich in H^+^. Hence, Cd was more prone to transport at the function of water, which made Cd reach a larger area in the PRD region in the soil forming process. Therefore, the Cd content in different soil layers had a high consistency.

#### Hg

The area of the PRD contaminated by Hg at concentrations above 0.5 mg kg^−1^was small (Fig 3B). Moreover, the contaminated area in deep soil was less than a third of that in the surface soil (Fig 4). Hg in the surface soil was distributed in areas where anthropogenic activity was intense, e.g., in Foshan and Guangzhou. The anomalies in the Hg distribution were likely related to human activity, such as industrial and agricultural production (e.g., [21]). Furthermore, the Hg released into atmosphere from anthropogenic point sources could have an impact on the terrestrial environments at the local, regional, and global scales [22,23]. For example, as a result of global-scale Hg deposition, it was estimated that the Hg concentrations in soil had increased by 15% [24]. According to Chen *et al*. [25], Guangdong, Foshan and Jiangmen are the most seriously Hg-polluted areas in the PRD due to their rapid economic and industrial development. According to Zhang *et al*. [26], anthropogenic activities have been the dominant source of the total emissions of Hg in the PRD. Among them, the municipal solid waste and coal combustion sources account for 28% and 21%, respectively. The production of cement and batteries also contributed substantially.

#### As

As, at concentrations above 20 mg kg^−1^, was widely distributed in the PRD; the areas of the surface soil and deep soil were 8507 km^2^ (accounting for 20.4% of the PRD) and 10040 km^2^ (accounting for 24.1% of PRD), respectively (Fig 4). The mean values of As in the surface (14.7 mg kg^−1^) and deep soil (16.5 mg kg^−1^) were larger than the national soil background (11.2 mg kg^−1^) [27]. Compared with studies in the coastal systems of British Columbia, the Meghna River Delta, and Hanoi area of Vietnam, the concentration of As in the sediment of the PRD was much higher because of its typical Quaternary sediments [28]. As in the sediment mostly came from terrestrial sources of bedrock outcrops in the PRD. There was abundant authigenic pyrite in the PRD, which incorporated a significant amount of As [29]. The main source of As in soil was its parent material as result of a series of processes among sedimentary, geochemical and biological factors. Meanwhile, Accumulation in metropolitan areas was most often attributed to fossil fuel combustion, particularly in coal, metal-processing industries, and mining activities (e.g., [30]). In the PRD, there is point-source pollution of As, resulting from anthropogenic activities, such as refining and mining [31,32].

#### Pb

The distribution of Pb (> 80 mg kg^−1^) in soil was randomly dispersed in the PRD, and there was no obvious regularity between the surface soil and deep soil (Fig 4). The mean value of Pb in the surface soil (42.3 mg kg^−1^), mainly distributed in Guangzhou and Foshan, was a little higher than in the soil background of the Guangdong Province (36 mg kg^−1^). These results indicated that instead of coming from parent material, Pb in the surface soil came from exogenous inputs. Guangdong and Foshan are developed areas with a well-established railway and highway transportation system, intense human activity, and a highly concentrated electronic industry. Previous studies have shown that the expansion of the road traffic infrastructure, especially in densely industrial and mining areas, has resulted in vehicle emissions contaminating the surrounding soil leading to changes in the original distribution of Pb [7,33,34].

Soils in downstream of the Xijiang and Beijiang River watershed were mainly Rhodic Paleudults, and those in the upstream and other areas around rivers were Typic Endoaquepts. Rhodic Paleudults and Plinthudults distributed in other areas of the PRD region. There was no obvious relationship between the soil type and heavy metals in the PRD region. There was a high level of Cd in the alluvial plain due to the long period of plain formation.

### Relationship between heavy metals in the surface soil and deep soil

**Fig 5:**
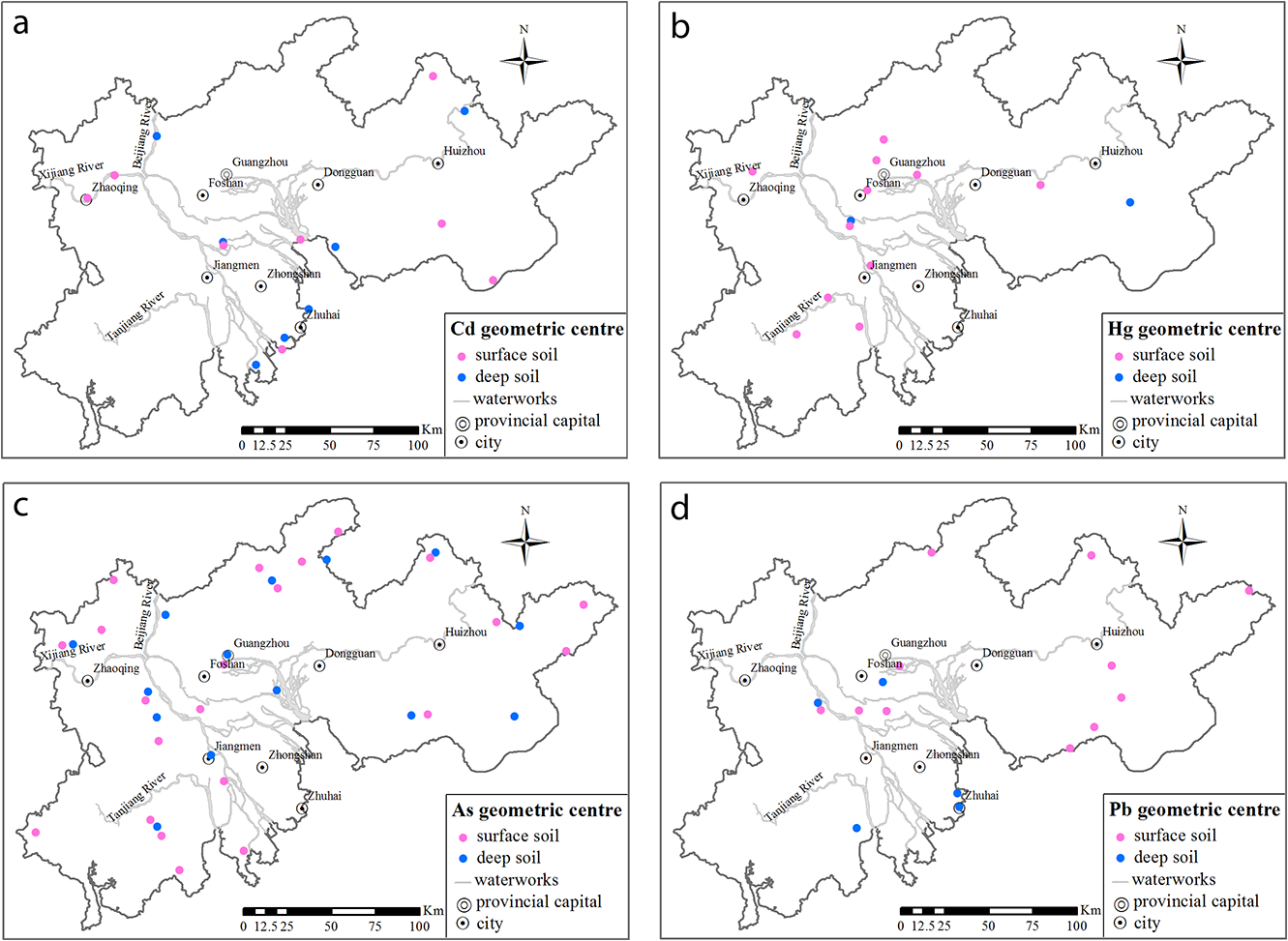
Geometric centers of PRD areas with high concentrations of heavy metals in the soil. (a) Cd; (b) Hg; (c) As; and (d) Pb.

**Fig 6:**
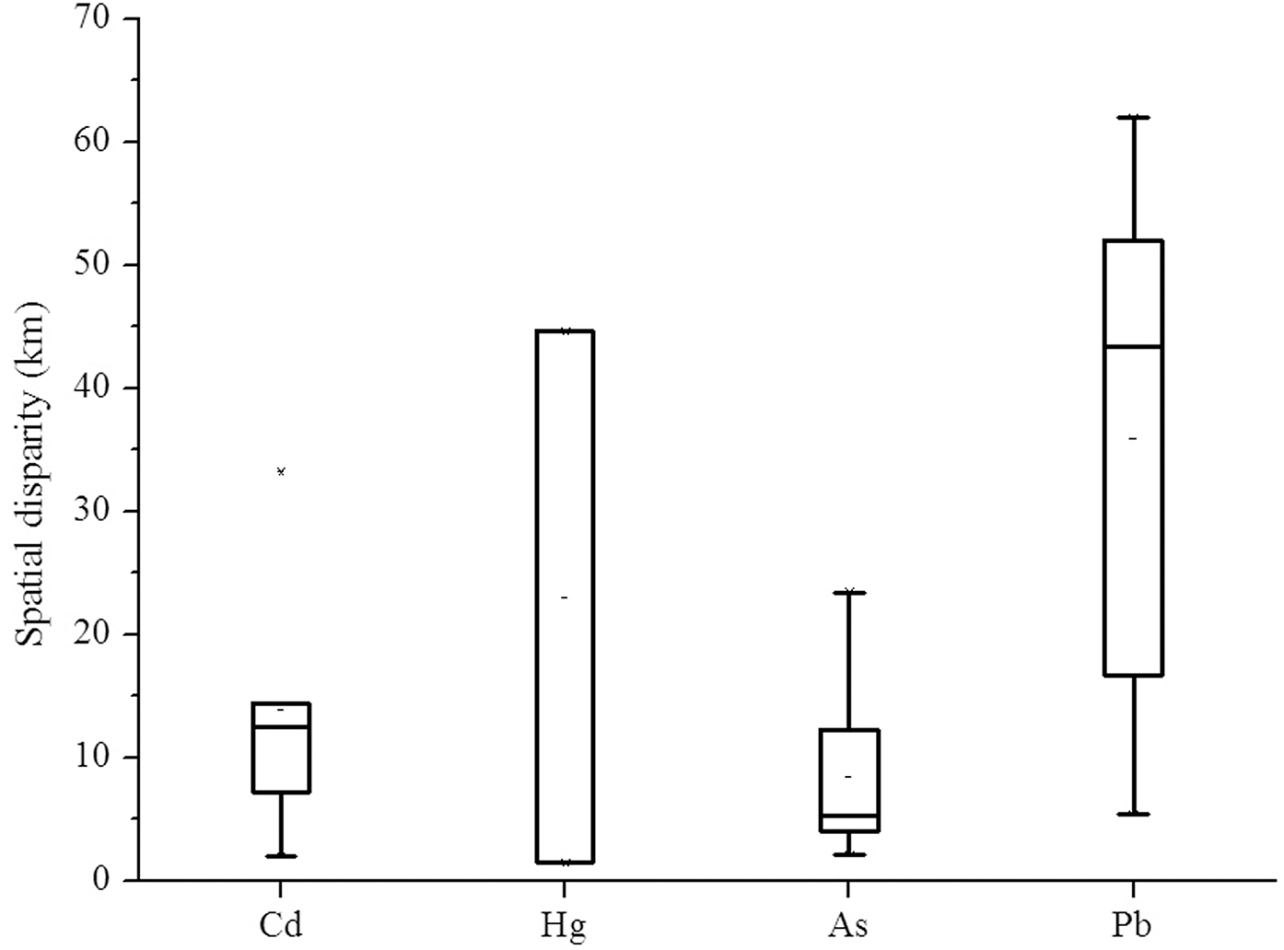
Spatial disparity of geometric centers in the surface and deep soil for heavy metals.

The spatial distribution in the surface and deep soil varied greatly for each heavy metal in the PRD. To assess the relationship of heavy metals presented in the top and deep soil, the geometric centers of the high-concentration areas (Figs 5 and 6) were analyzed.

The largest *d* value was observed for Pb (35.9 km), which was followed by Hg (23.0 km), Cd (13.8 km) and As (5.1 km). The correlation between the surface and deep soil for Cd and As was larger than that for Pb and Hg. The spatial disparity of Cd ranged from 2.0 to 14.4 km, with an outlier of 33.2 km. As was similar to Cd, but showed less diversity. Although the spatial disparities of Cd and As were similar (Fig 6), they were essentially different based on the results presented in section 3.1. For the former, there was because of the migration of Cd along surface water, in the long soil forming process of suspended solids deposition in the watershed of the Xijiang and Beijiang Rivers. The latter had a high background value. In the case of Hg and Pb, there was notable diversity as well as little association between the surface and deep soil. This further indicates that exogenous inputs were the dominant sources of Hg and Pb in the PRD region.

### Potential ecological risk assessment

**Fig 7:**
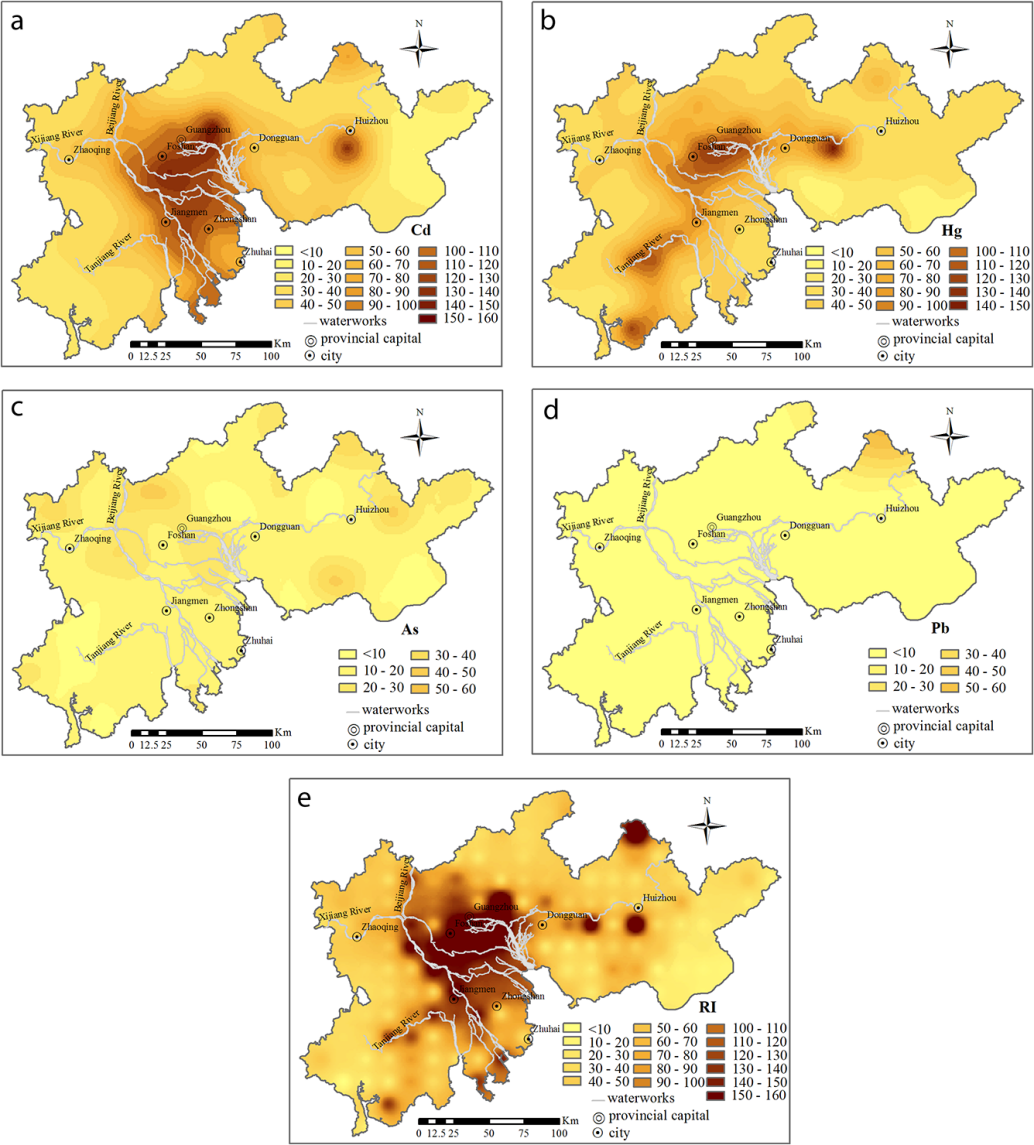
Potential risk index (RI) of heavy metals in the soil of the PRD. (a) Cd; (b) Hg; (c) As; (d) Pb; and (e) the four heavy metals.

The Håkanson index provides a quantitative method for directly isolating the extent of potential hazards. The potential ecological risk index and single heavy metal contamination index yielded different assessment results (Fig 7).

In terms of single-factor pollution, Cd, Hg, As and Pb were all at the low ecological risk level. The high-risk areas for Cd were mainly located in the Beijiang and Xijiang River (Fig 7A), and there were areas along rivers of higher Hg risk (Fig 7B) and low toxicity risks for As and Pb. The Cd pollution in the PRD had a long history because of the equally long presence of its relevant outputs as well as because the geographical conditions were conducive to its transportation. There have been several pollution emergencies related to Cd in the tributaries of the Pearl River reported since 2000, all of which have had serious impacts on environmental health [3]. Despite the relatively high ecological risk of Hg in the PRD, its concentration was not high in the surface soil or deep soil (Fig 3B).

The spatial distribution of the potential ecological risk index in the surface soil of the PRD indicates that areas that were previously identified by different indices as potentially polluted areas represented the greatest ecological risk. The distribution patterns of the individual Cd potential ecological risk indices were almost the same as those of the general potential ecological risk indices for all heavy metals. This indicated the important contribution of Cd to the general indices. Moreover, area iii (Fig 7E) also had higher ecological risk, which might be due to its location of the potential prospecting areas with polymetallic ore [16]. Anomalous values of tungsten coincide with many associated ores, which might increase the ecological risk in the processes of mineral exploitation and smelting. In general, the single potential ecological risk indices indicated that the severity of pollution with respect to the four heavy metals decreased in the following order: Cd ≥ Hg > Pb > As.

Therefore, the variability of Cd in the study area should be controlled on a regional scale [35], especially for the upper reaches of the Beijiang and Xijiang Rivers. In areas with intense human activity, such as Foshan and Guangzhou City, proper measures should be taken to control the industrial emissions. For high background-value areas, anthropogenic disturbance such as mining and refining might increase the risk to ecology and human health. However, this balance could be maintained. For example, although the concentration of As was high in the PRD, it might be possible to maintain an ecological balance if some measures are taken with respect to mineral exploration, such as using cleaning production techniques to reduce/eliminate the risk from the environmental risk source and controlling mechanism.

## Conclusions

The present study revealed a diverse range of distribution areas and trends for heavy metals because of various sources in the PRD region. As and Cd were more associated with natural processes than Hg and Pb. Cd mainly diffused around the Xijiang and Beijiang River watershed while As was rich in the parent material. The ecological risk was mainly attributed to the migration of Cd. Single potential ecological risk indices indicated that the severity of pollution decreased in the following order: Cd ≥ Hg > Pb > As. The pollution of Hg and Pb due to anthropogenic activities should be controlled in the local area because of point source pollution. For As, the control of cleaning production could reduce/eliminate the environmental impact.

## Supporting Information

S1 Fig Geochemical map of Cadmium in surface soil. (TIF)

S2 Fig Geochemical map of Cadmium in deep soil. (TIF)

S3 Fig Geochemical map of Mecury in surface soil. (TIF)

S4 Fig Geochemical map of Mecury in deep soil. (TIF)

S5 Fig Geochemical map of Arsenic in surface soil. (TIF)

S6 Fig Geochemical map of Arsenic in deep soil. (TIF)

S7 Fig Geochemical map of Lead in surface soil. (TIF)

S8 Fig Geochemical map of Lead in deep soil. (TIF)
