# Supplementary figures and images for: The Source, Spatial Distribution and Risk Assessment of Heavy Metals in Soil from the Pearl River Delta Based on the National Multi-Purpose Regional Geochemical Survey
Source: PLoS One. 2015 Jul 31;10(7):e0132040. doi: 10.1371/journal.pone.0132040 (PMC4521848; doi:10.1371/journal.pone.0132040)

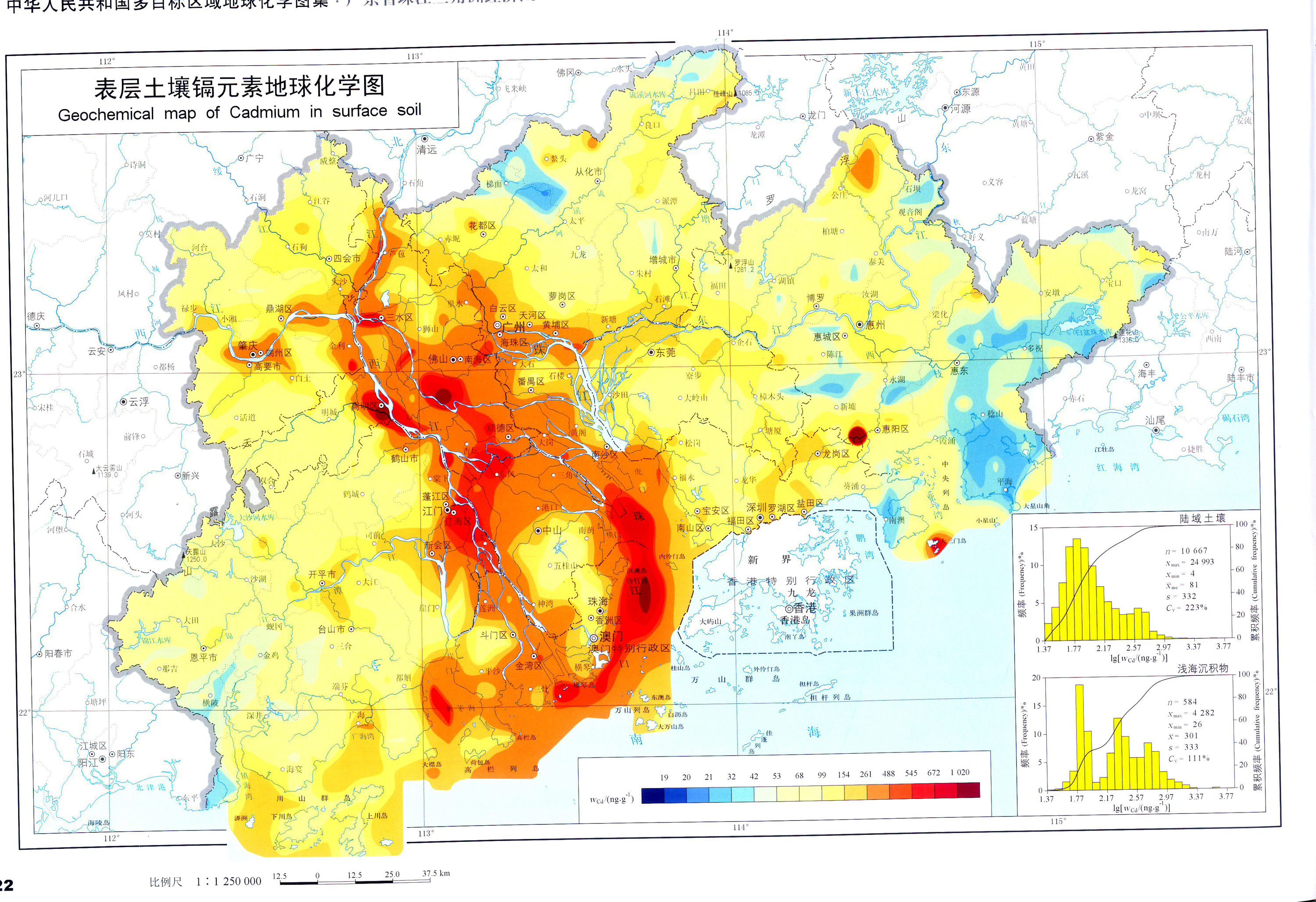

Supplement: S1 Fig — (TIF) [file pone.0132040.s001.tif]

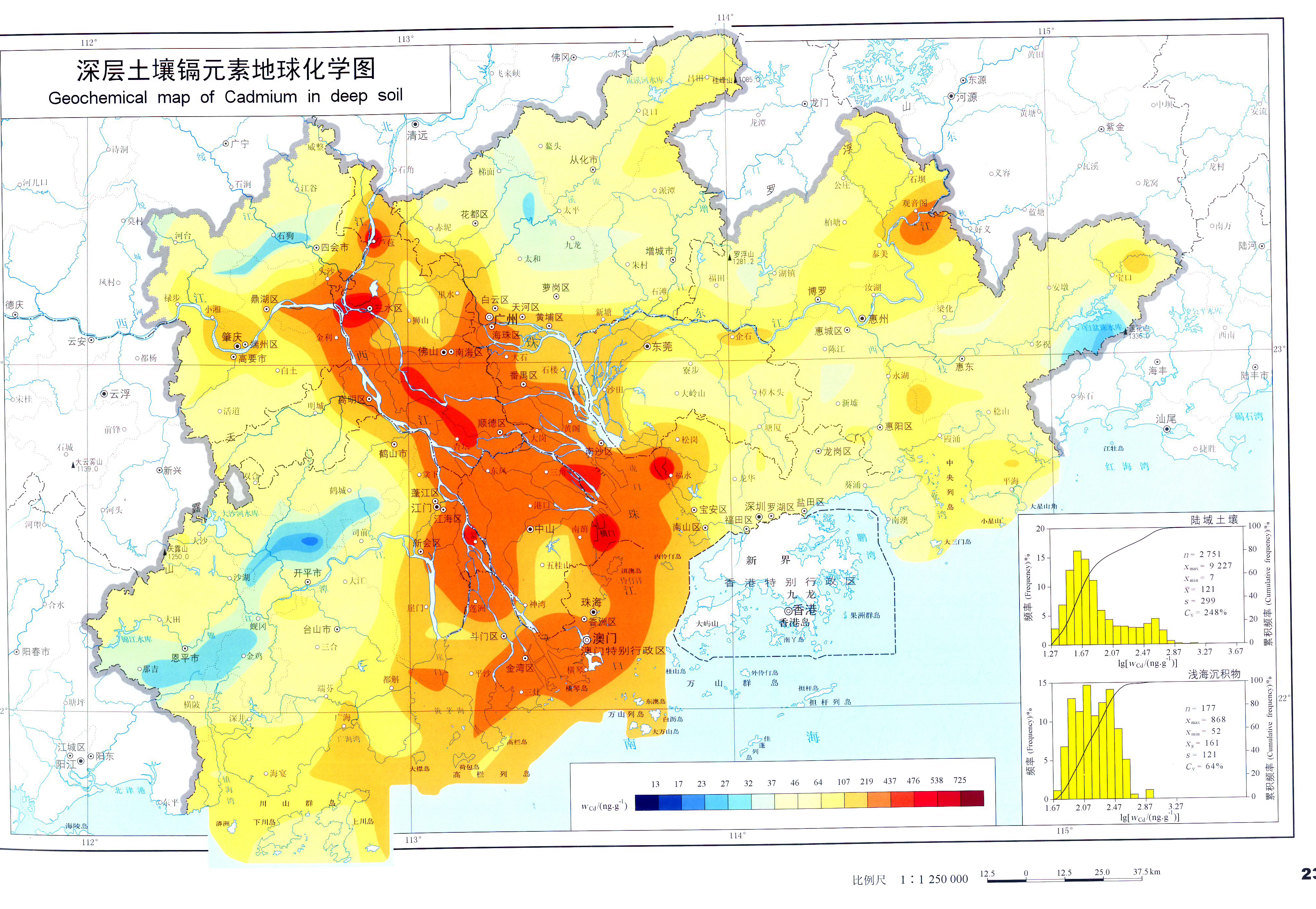

Supplement: S2 Fig — (TIF) [file pone.0132040.s002.tif]

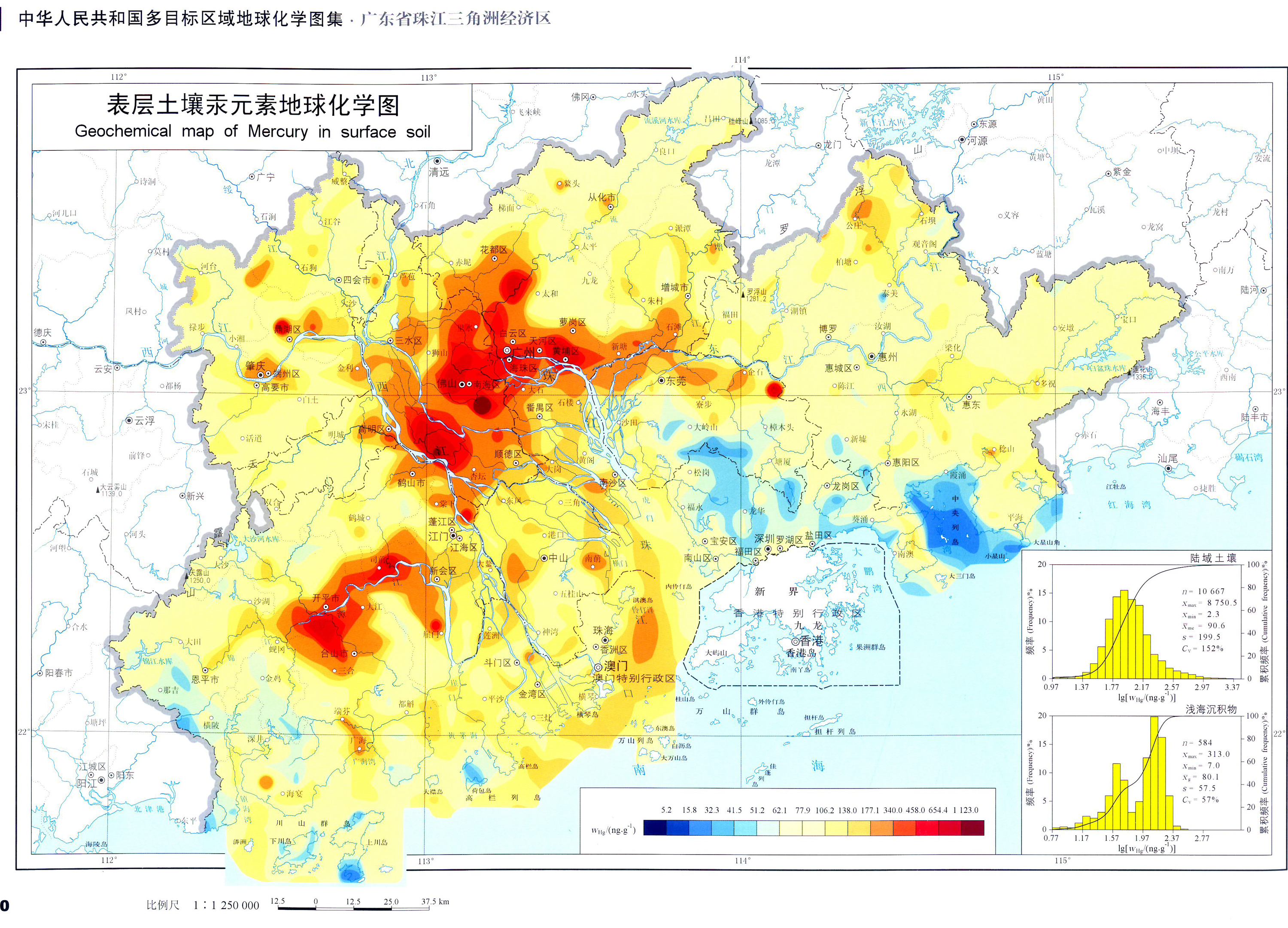

Supplement: S3 Fig — (TIF) [file pone.0132040.s003.tif]

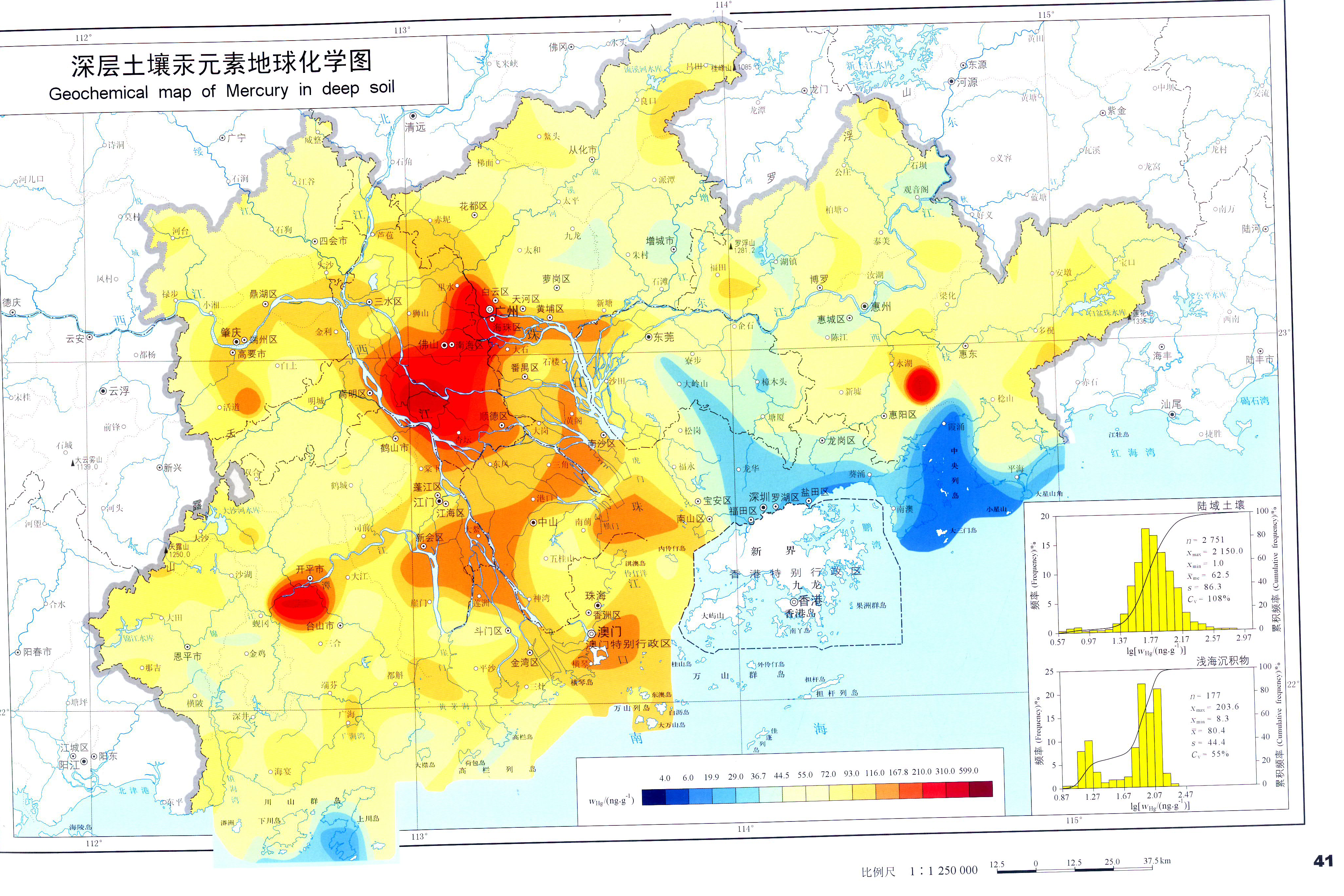

Supplement: S4 Fig — (TIF) [file pone.0132040.s004.tif]

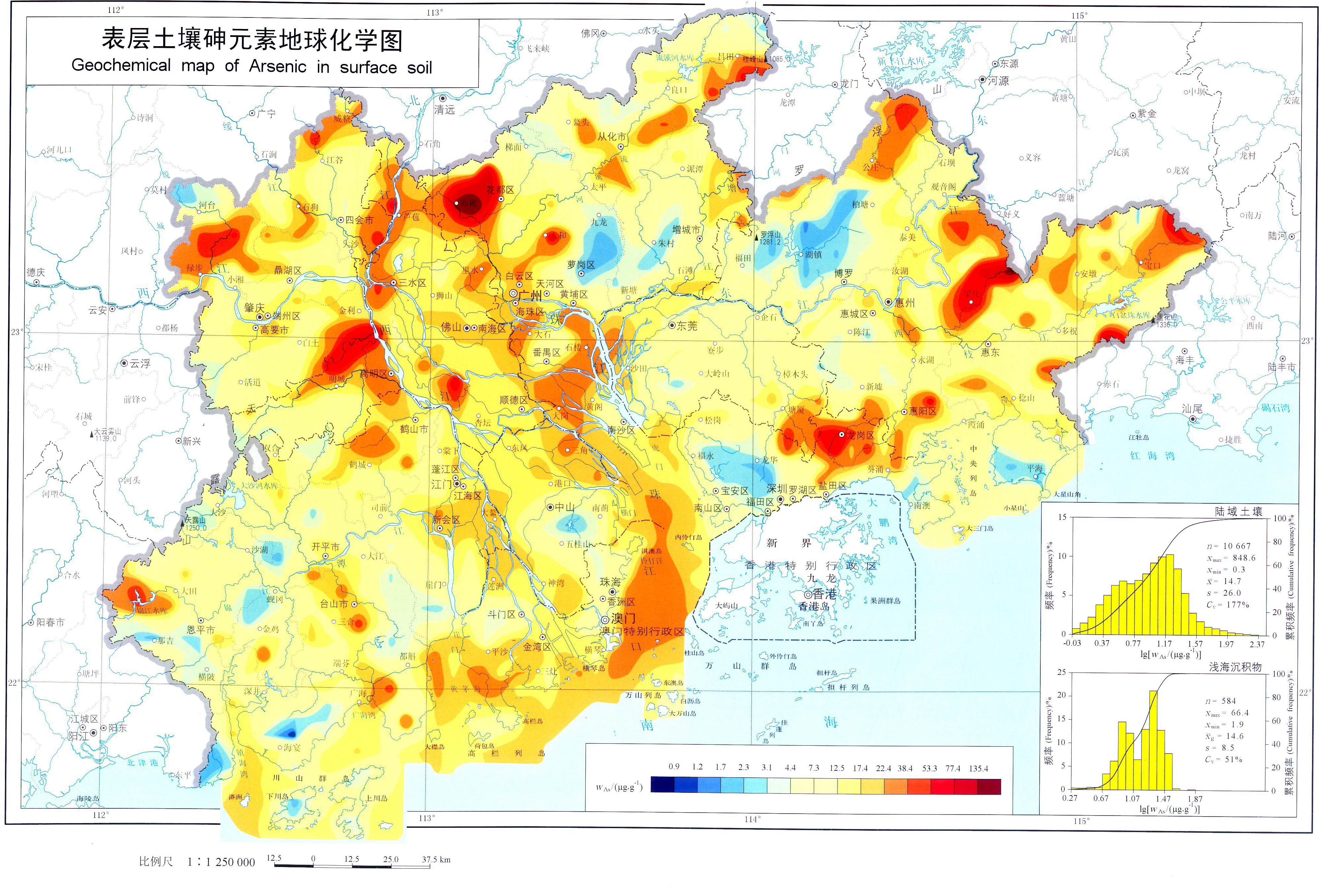

Supplement: S5 Fig — (TIF) [file pone.0132040.s005.tif]

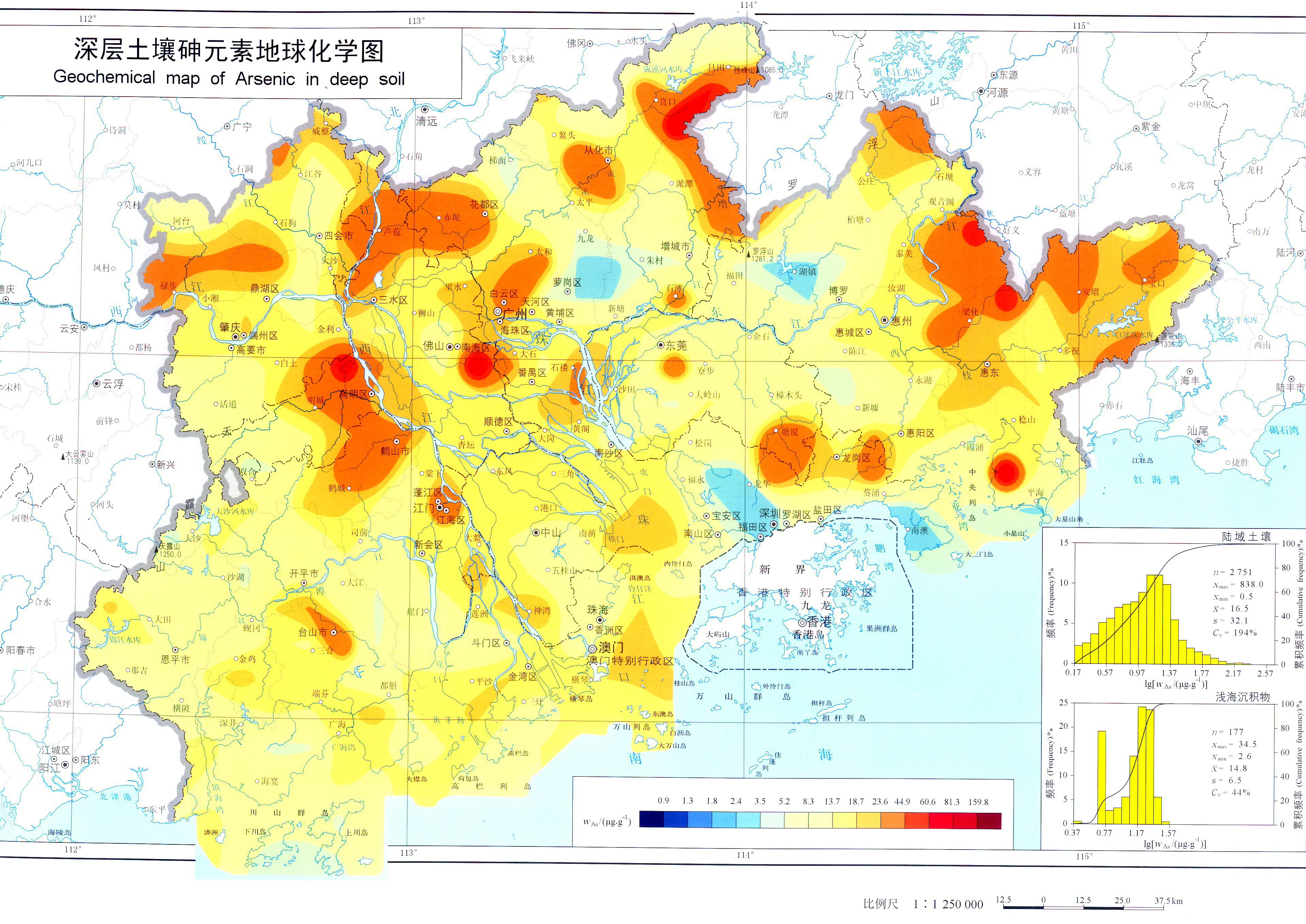

Supplement: S6 Fig — (TIF) [file pone.0132040.s006.tif]

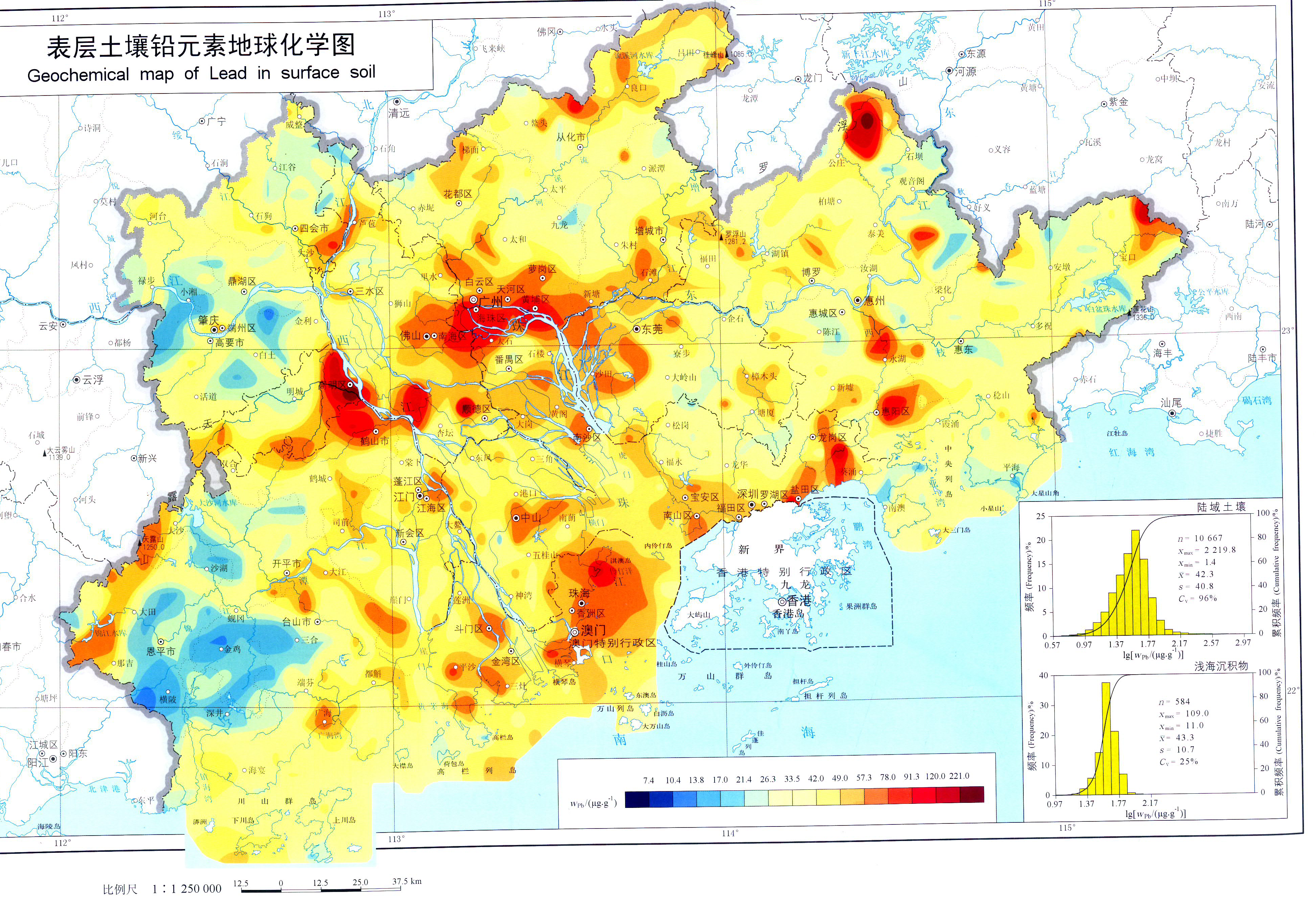

Supplement: S7 Fig — (TIF) [file pone.0132040.s007.tif]

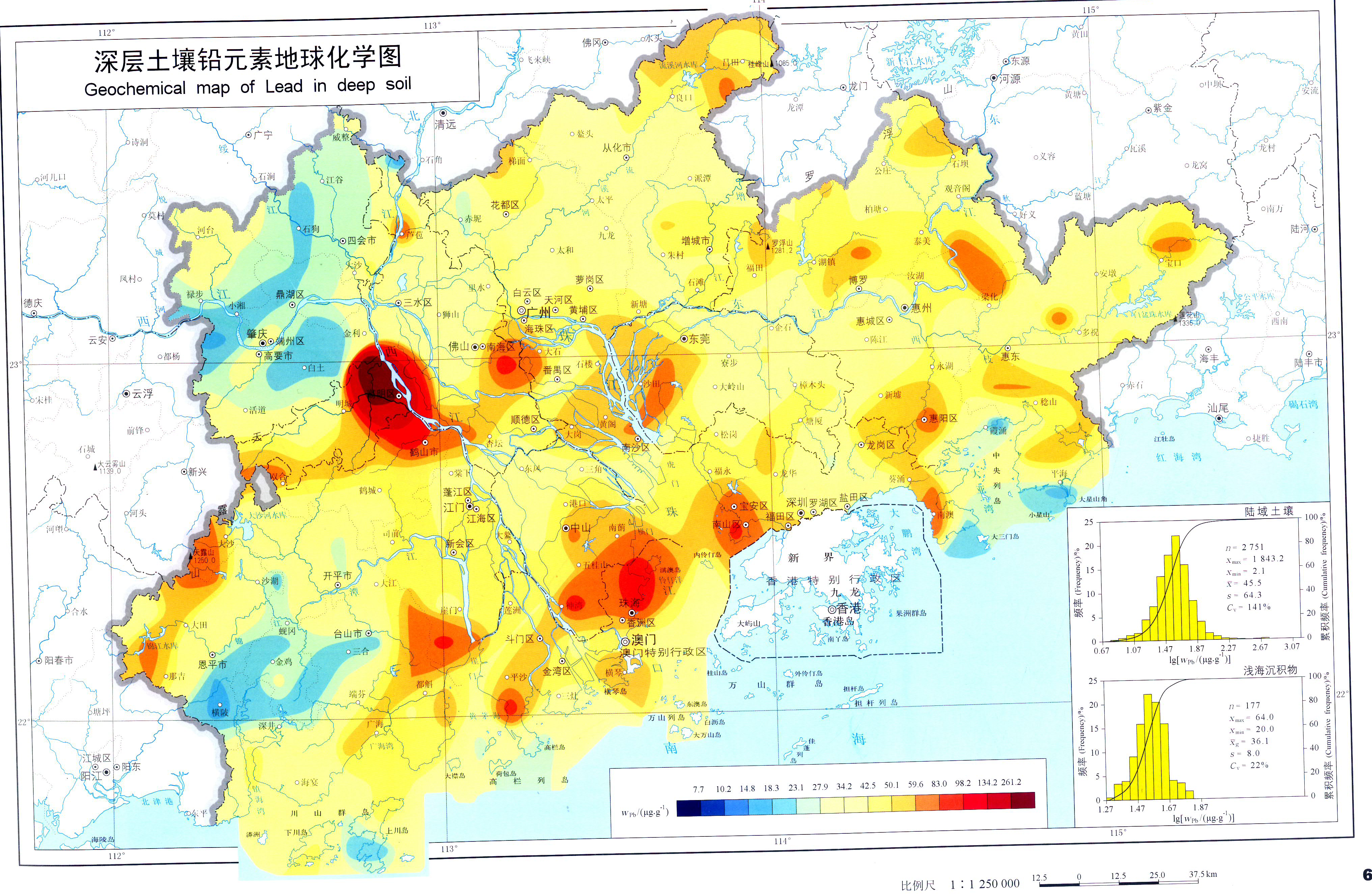

Supplement: S8 Fig — (TIF) [file pone.0132040.s008.tif]
